# Validation of the Lean Healthcare Implementation Self-Assessment Instrument (LHISI) in the finnish healthcare context

**DOI:** 10.1186/s12913-021-07322-2

**Published:** 2021-12-01

**Authors:** Elina Reponen, Ritva Jokela, Janet C. Blodgett, Thomas G. Rundall, Stephen M. Shortell, Mikko Nuutinen, Noora Skants, Markku Mäkijärvi, Paulus Torkki

**Affiliations:** 1 Center for Lean Engagement and Research in Healthcare, School of Public Health, University of California, Berkeley, California USA; 2 HUS Helsinki University Hospital, P.O.Box 760, 00029 Helsinki, Finland; 3 Haartman Institute, University of Helsinki, Helsinki, Finland; 4 Department of Public Health, University of Helsinki, Helsinki, Finland

**Keywords:** Lean healthcare, Lean management, Lean implementation, Self-assessment instrument, Lean maturity assessment

## Abstract

**Background:**

Lean management is growing in popularity in the healthcare sector worldwide, yet healthcare organizations are struggling with assessing the maturity of their Lean implementation and monitoring its change over time. Most existing methods for such assessments are time consuming, require site visits by external consultants, and lack frontline involvement. The original Lean Healthcare Implementation Self-Assessment Instrument (LHISI) was developed by the Center for Lean Engagement and Research (CLEAR), University of California, Berkeley as a Lean principles-based survey instrument that avoids the above problems. We validated the original LHISI in the context of Finnish healthcare.

**Methods:**

The original HISI survey was sent over a secure organizational email system to the over 26,000 employees of the Hospital District of Helsinki and Uusimaa in March 2020. The data were randomly split with one part used to carry out an exploratory factor analysis (EFA), and the other for testing the resulting model using confirmatory factor analysis (CFA).

**Results:**

A total of 6073 employees responded to the LHISI survey, for an overall response rate of 23%. The results indicated that the 43 items used in the original LHISI can be reduced to 25 items, and these items measure a five-dimensional model of the progress of Lean implementation: leadership, commitment, standard work, communication, and daily management system. In comparison with a single-factor model, the fit measures for the 5-factor model were better: smaller X^2^, larger comparative fit index (CFI), smaller root mean square error of approximation (RMSEA), and smaller standardized root mean square residual (SRMR).

**Conclusions:**

The 25 item LHISI is valid and feasible to use in the context of Finnish healthcare. The LHISI allows the organization to self-monitor the progress of its Lean implementation and provides the leadership with actionable knowledge to guide the path towards Lean maturity across the organization. Our findings encourage further studies on the adoption and validation of the LHISI in healthcare organizations worldwide.

**Supplementary Information:**

The online version contains supplementary material available at 10.1186/s12913-021-07322-2.

## Background

Many healthcare organizations worldwide have implemented Lean management practices to improve hospital performance and patient outcomes. Lean can be defined as a management system that promotes a continuous improvement culture that empowers front line workers (nurses, physicians, support staff) to solve problems and eliminate waste by standardizing work to increase the value of care delivered to patients [1]. It is a socio-technical system approach emphasizing culture, leadership, work design, and a set of techniques to support organization performance improvement [2–5]. The implementation of Lean philosophy, cultural values, tools and behaviors is a transformational journey that is influenced by many characteristics of the organization including leadership commitment, managerial and clinical support, and resources for staff training. Furthermore, Lean applications are highly variable across organizations [6, 7]. Within a given healthcare organization, Lean may be more easily implemented in some units than others resulting in considerable fragmentation [8–10]. Consequently, while some healthcare organizations have reached Lean maturity, many struggle to move beyond the start-up stage [1].

This heterogeneity and the multidimensionality of Lean suggest that hospital leaders would benefit from periodically conducting a Lean implementation assessment to understand the extent of Lean implementation in their organization. This will help them in identifying areas in which to focus attention. Guimarães and Carvalho, for example, noted the importance of this type of assessment to successful organization transformation: “Lean deployment assessment in healthcare is needed to understand the depth of Lean deployment, avoid misconceptions of Lean, and guide health care organizations in pursuing a new management philosophy rather than a fad. Most Lean change attempts lack monitoring and continuous double-loop learning, leading to returns to the comfort zone and, therefore, to the absence of Lean sustainability” [11]. Many assessment instruments exist, but have a number of limitations, including: incomplete coverage of Lean principles; intended for use by an external consultant rather than hospital staff; excessive length; and the requirement that the questions in the instrument be answered only in the context of a site visit by expert assessors. These limitations make regular use of the instrument operationally infeasible for many organizations.

Addressing this gap in the field, The Center for Lean Engagement and Research in Healthcare (CLEAR) at the School of Public Health, University of California, Berkeley, used an iterative approach to develop the Lean Healthcare Implementation Self-Assessment Instrument (LHISI) with an aim to provide healthcare organizations with a feasible, principles-based tool for assessing their Lean maturity [12]. The data used for the development and validation of the LHISI were derived from four Lean hospitals in the US: two university medical centers, a public hospital in an urban area, and a community hospital in a rural region. However, Lean healthcare organizations around the world face similar challenges in measuring their Lean maturity. Context may influence the prerequisites and conditions for Lean implementation, but the applicability and validity of the LHISI instrument in healthcare organizations operating outside the US remains undefined. The aims of our study were:

1. to assess the applicability and validity of the original LHISI in a large academic hospital system in Helsinki, Finland.
2. to evaluate whether the LHISI survey covers key Lean elements and offers actionable information to the management.

## Methods

**Fig. 1:**
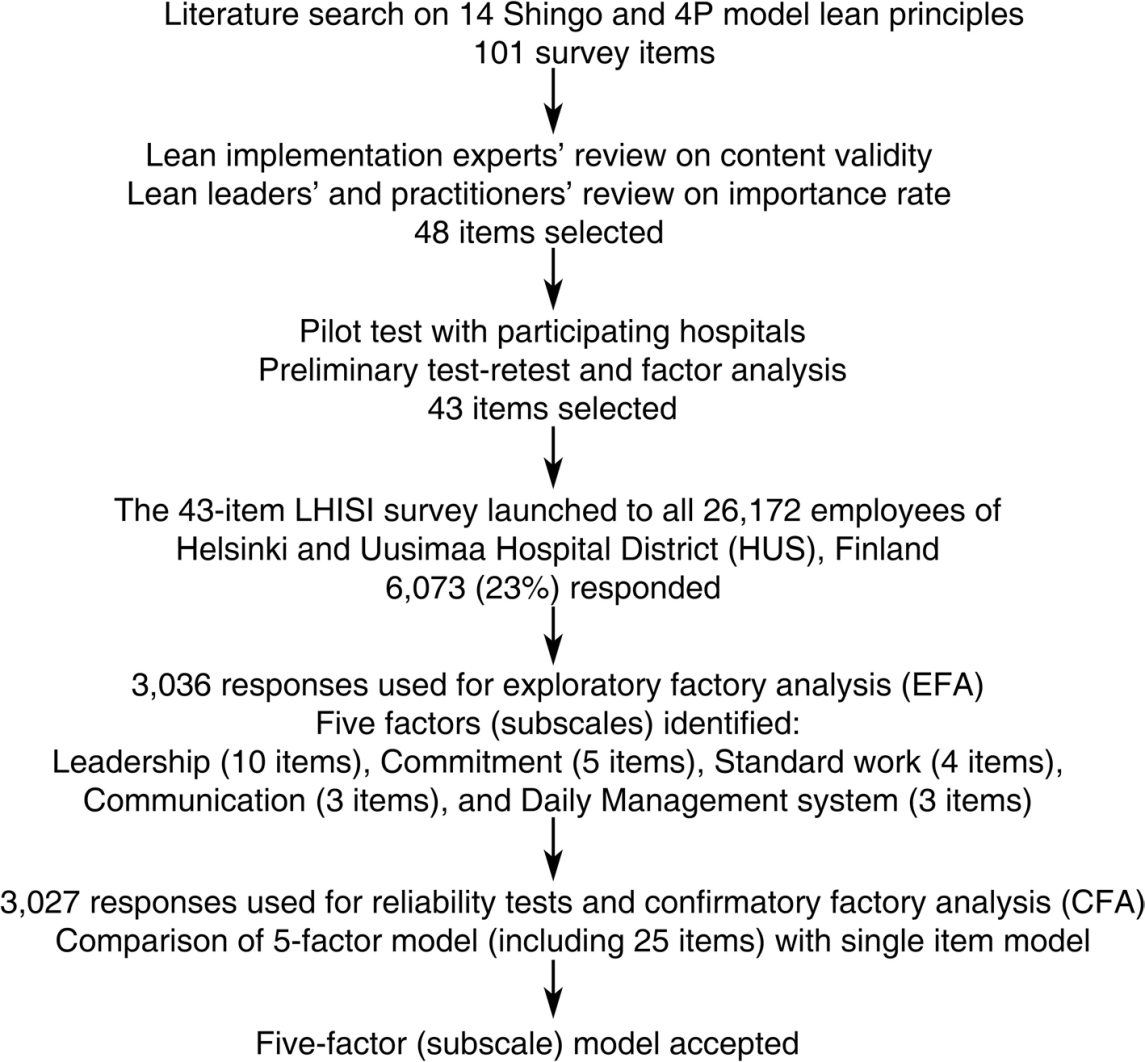
Development of the Lean Healthcare Implementation Self-assessment Instrument (LHISI)

The initial development of the original LHISI was completed as part of a Lean healthcare research learning collaborative managed at the University of California Berkeley, involving data from several hospitals and health systems in the United States. First, a list of 101 potential survey items (comprising specific Lean-related actions) was developed based on 14 principles of Lean management outlined in the Shingo and 4P models [2, 3]. Several steps were taken to create an instrument that would fulfill the prerequisites for a high-quality maturity assessment tool: validity, feasibility (manageable length, self-assessment), relevance and reliability of the results. Lean implementation experts assessed the content validity of the items, and Lean leaders and practitioners rated the importance of each item in measuring the extent of hospital Lean implementation, reducing the number of potential survey items to the 48 most important items. There was a 9-level response scale for each item, with three anchors (0 = Never, 4 = Sometimes, 8 = Always). The 48-item instrument was pilot tested with the participating hospitals, and preliminary test-retest and factor analyses were completed. This process identified an additional five items that had limited reliability or did not clearly contribute to a factor, producing a 43-item instrument that was tested in the current study (Additional file 1). The development of the original 43 item LHISI instrument is depicted in Fig. 1.

The 43-item LHISI survey was sent to all 26,172 employees of Hospital District of Helsinki and Uusimaa (HUS) in March 2020. HUS is the largest of five academic health systems in Finland. It provides secondary and tertiary care for a population of 2.2 million, and quaternary care (care for severe and uncommon diseases) for all residents in Finland. All professionals who work in HUS, including the doctors, are HUS employees. We translated the LHISI into the two official languages in Finland, Finnish and Swedish. The Lean experts of the HUS Lean development unit worked together with in-house official language translation services to ensure the integrity of the translated version. We used an internet-based survey platform (Vibemetrics Ltd.), and responses were anonymous. Background information for each respondent included unit, professional group (doctor, nurse, support staff), and years working in HUS. The survey was open for responses for 2 weeks and the number of responses was continuously monitored. After the initial email inviting the employees to participate, three reminders were sent eight days, five days, and one day before the survey was closed.

The full anonymized dataset was randomly split into two parts: one used to carry out an exploratory factor analysis (EFA), and one testing the resulting model using confirmatory factor analysis (CFA). A comparison of the background characteristics (respondents’ employee group, years working in the organization, and department) revealed no significant differences between the two samples created through random assignment. All analyses were completed using R version 4.0.2 [13, 14].

First, we identified potentially redundant measures by examining all item-pair Pearson correlations greater than .8, and selected one of each pair for removal from the final model. Next, we carried out the exploratory factor analysis using functions provided in the psych package [15]. We used principle axis factor extraction because we did not assume multivariate normality [16]. We assumed that the factors that would emerge from our analysis would reflect distinct dimensions of the construct of overall lean implementation, and that underlying characteristics of hospital units and personnel would likely influence multiple dimensions, making the extracted factors likely to correlate with each other, so we used oblimin rotation [16, 17]. To assess the appropriateness of choosing the oblimin rotation based on expected item correlation, we tested the varimax rotation commonly used in EFA, which constrains the correlations to zero. Using the varimax rotation took 25 iterations before any meaningful data was produced confirming that trying to constrain the items to be uncorrelated was inappropriate. Parallel analysis suggested the initial number of factors to extract, and we ensured that all factors in this first extraction had at least 3 items with loadings >.3 as suggested by Costello and Osborne [16]. We reduced the number of extracted factors by one at a time until this criterion was met. Next, we examined all factor loadings, aiming to produce a final model with each item loading on a single factor > .4 (a common cutoff used in published factor analyses) [18]. In addition, to better approximate a simple structure (each item loading on only one factor), we required that items did not cross-load on multiple factors with a loading difference of ≤.2 from the highest loading. This is within the magnitude suggested by Worthington and Whittaker (2006) [18]. If cross-loading was present, we removed the item with the smallest high loading, and repeated the analysis.

We used the second data set to test the final model created in the EFA. We calculated reliability coefficients (Cronbach alpha) for each factor, and measured the correlation between the factors. Next, we carried out a CFA (using the lavaan package) [19] to compare fit measures for the final model to those of a model with all items loading on a single factor [20, 21].

To summarize survey results on the unit level, the individual survey items were grouped into subscales according to the results of the factor analyses. The subscale score for each unit was assigned by calculating a weighted average of the ratings of the items within each subscale, weighted by the factor loading values. Each subscale had a possible range of 0 to 8. First, a subscale score for each respondent was calculated by taking the weighted average of available ratings across items within each subscale. The subscale score was only calculated if the respondent had answered at least 50% of the items included in that subscale. “I don’t know” response was considered a missing value for the purposes of subscale score calculation. Finally, the unit-level subscale was calculated as the arithmetic mean of the individual subscale scores within each unit.

## Results

Of the 26,172 employees invited to participate 6073 responded yielding a response rate of 23%. 3036 of the responses made up the training dataset used for the EFA. The minimum number of pairwise comparisons, i.e., submissions with a response to both items in the pair, in the training dataset (across the 43 items, a total of 903 pairs) was 1066 observations (mean = 1904, max = 2892). Seven items were selected for removal from further analysis due to high (>.8) item-pair correlation (see Additional file 2). Limited to the remaining 35 items, parallel analysis suggested an 8 factor model. The initial analysis did not have at least three items with loadings >.3 on each factor, and after lowering the number of extracted factors one at a time until this was true, the resulting model included five factors. Next, we looked for cross loading and removed one item at a time until the factor structure included each item loading on a single factor > .4, with no cross-loading within .2. Additional file 2 shows the further 11 items removed at this stage, and Table 1 shows the final factor structure and eigenvalues (all are > 1), loadings greater than 0.4, and commonalities (h^2^). All commonalities are above 0.5, indicating that the factors do a good job of explaining variance within the retained items.

**Table 1:** Final exploratory factor analysis loadings and factor eigenvalues

Item	Leadership	Commitment	Standard work	Communication	Daily Management System	h2
Eigenvalue	5.59	4.05	3.06	2.48	2.44	
q09. Across my hospital/clinic, leaders at all levels create a safe environment for exposing problems.	0.762					0.705
q10. Across my hospital/clinic, senior leaders practice humble inquiry when interacting with employees at all levels of the organization.	0.924					0.794
q11. Across my hospital/clinic, leaders at all levels engage employees where the work happens.	0.808					0.686
q13. Across my hospital/clinic, leaders at all levels create and sustain an environment of continuous improvement and continuous learning.	0.659					0.767
q16. In my unit/department, senior leaders have made an explicit commitment to patient-centered care.	0.563					0.576
q29. In my unit/department, senior leaders follow a process for strategy definition and deployment that provides focus at all levels.	0.586					0.754
q36. Across my hospital/clinic, leaders at all levels coach to ensure a clear connection between purpose and the work being performed.	0.548					0.744
q37. Across my hospital/clinic, leaders at all levels provide employees and staff regular feedback.	0.677					0.63
q40. In my unit/department, senior leaders make data driven decisions.	0.432					0.621
q43. Across my hospital/clinic, successes gained and failures are shared.	0.407					0.538
q02. In my unit/department, management staff use PDSA thinking with the operational units they lead.		0.696				0.643
q06. In my unit/department, management staff are committed to lean.		0.862				0.774
q07. In my unit/department, physicians are committed to lean.		0.752				0.677
q12. Lean has a sponsor/champion and clinical and management staff who demonstrate visible, active, public commitment and support of lean.		0.76				0.816
q14. In my unit/department, management staff practice A3 thinking.		0.753				0.751
q21. In my unit/department, use of standard work is monitored for compliance.			0.732			0.753
q22. In my unit/department, clinical staff use standard work.			0.809			0.8
q24. In my unit/department, senior leaders use standard work.			0.54			0.78
q25. In my unit/department, work processes are standardized.			0.607			0.675
q26. In my unit/department, those who provide care to patients/customers communicate with each other.				0.771		0.672
q27. In my unit/department, the communication that occurs among those who provide care to patients/customers is focused on problem-solving rather than blaming each other or others.				0.767		0.68
q28. In my unit/department, those who provide care to patients/customers share common goals.				0.782		0.751
q32. In my unit/department, clinical staff attend daily huddles.					0.88	0.75
q33. In my unit/department, management staff attend daily huddles.					0.736	0.642
q38. In my unit/department, a daily management system (e.g., daily huddles, gemba walks, etc) is used.					0.618	0.656

Factor loadings <.4 have been suppressed. Question numbering follows the numbering in the original survey (Additional file 1)

The first of the five factors included ten survey items that address leader qualities, attitudes, and activities, and was thus named “Leadership”. The second factor was named “Commitment” and it comprises five survey items that address issues around management and staff discipline and commitment. The third factor, “Standard work” included four survey items addressing the existence, use, and monitoring of standard work. The fourth and fifth factors included three items each. The items in the fourth factor “Communication” addressed issues around collaboration and communication, and the fifth factor was named “Daily Management System” with items addressing the daily management system and its elements such as daily huddles and gemba walks. The individual items included in each of the five factors are presented in Table 1. In practical applications of the LHISI results, the factors are called subscales.

We applied the 5-factor structure suggested in the EFA to the second half of the data, comprising 3027 survey responses. Table 2 summarizes the factor score mean for each factor, Cronbach’s alpha measuring internal consistency, and the factor correlation scores. The factors are well correlated, supporting the idea that they are related and measuring aspects of an underlying concept (overall extent of hospital lean implementation). The CFA fit measures are shown in Table 3, comparing the proposed 5-factor model to a model loading all items onto a single factor. The 5-factor model had a better fit by all measures. Furthermore, the 5-factor model had a better fit by all measures compared to the 6-factor model constructed during earlier stages of LHISI development (Additional file 4). The distribution of factor loadings indicated high measurement quality (mean = 0.817, median = 0.827, min = 0.693, max = 0.893). In such cases, traditional rules of thumb for common fit statistics may be too conservative [18]. In comparison with the single-factor model, the fit measures for the 5-factor model were better: smaller X^2^, larger comparative fit index (CFI), smaller root mean square error of approximation (RMSEA), and smaller standardized root mean square residual (SRMR). The SRMR of the 5-factor model, 0.05, is below the traditional rule of thumb (<.08) [18]. The CFI and RMSEA values (0.921 and 0.068 respectively) are slightly outside of Hu and Bentler’s rules of thumb (>.95 and < .06), [18] but fall within the range suggested by McNeish et al. [19]. Additional file 3 presents the 25 items included in the final, validated LHISI (version 3.0).

**Table 2:** Confirmatory factor analysis: summary statistics

Factor	Factor Score Mean (SD)	Cronbach alpha	Factor correlations				
			Leadership	Commitment	Standard work	Communication	Daily Management System
**Leadership (10 items)**	2.43 (1.01)	0.948	1				
**Commitment (5 items)**	4.2 (2.08)	0.932	0.822	1			
**Standard Work (4 items)**	5.38 (1.88)	0.922	0.732	0.732	1		
**Communication (3 items)**	6.28 (1.47)	0.87	0.613	0.54	0.695	1	
**Daily Management System (3 items)**	5.02 (2.22)	0.825	0.617	0.685	0.629	0.503	1

**Table 3:** Confirmatory factor analysis: Fit index comparison

Fit index	5-factor model	Single-factor model
X^2^ (df), *p*-value	4013.756 (265), 0	28,154.846 (860), 0
CFI	0.921	0.705
RMSEA	0.068	0.102
SRMR	0.05	0.073

*CFI* comparative fit index, *RMSEA* root mean square error of approximation, *SRMR* standardized root mean square residual

**Fig. 2:**
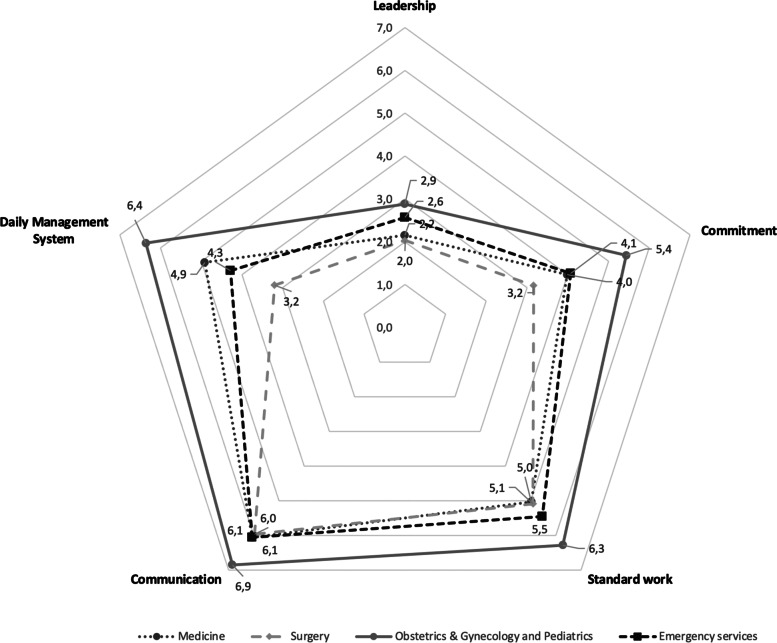
LHISI survey results in HUS Hyvinkää Hospital Area clinical departments

Across all clinical and support services in HUS, subscale scores were reported for 47 departments with at least 5 responses to maintain anonymity. Subscale scores varied from 2.01 to 3.48 for Leadership, from 3.16 to 5.89 for Commitment, from 3.77 to 6.73 for Standard work, from 3.67 to 7.67 for Communication, and from 2.67 to 6.36 for Daily Management System. Figure 2 demonstrates how the validated LHISI subscale score results can be illustrated with a spider web chart, using HUS Hyvinkää Hospital Area clinical departments as an example.

## Discussion

The EFA and CFA results indicate that the 25 item LHISI instrument is valid in the Finnish healthcare context. The five factors identified through the factor analyses – Leadership, Commitment, Standard work, Communication, and Daily Management System – are all key components of Lean management indicating that the principles-based approach in developing the LHISI is reflected in the survey results. Using the factors as subscales and calculating subscale scores separately enables a more detailed understanding of the progress of Lean transformation in a healthcare organization or a specific unit and interrelationships of Lean domains. Our findings encourage more studies in healthcare organizations around the world to translate and validate the LHISI instrument in other countries. Worldwide use of the LHISI would facilitate benchmarking the maturity of Lean in general and its individual components both locally and internationally.

Several instruments measuring the progress or the extent of Lean implementation exist, but they have been developed in and designed for the manufacturing industry [11]. Some of these instruments are survey-based and structurally similar to the LHISI: the survey is conducted among employees, and the aim is to measure different dimensions of Lean using typically between 20 and 40 survey items [22–24]. The instrument developed by Loyd and coworkers, for example, in the manufacturing industry is validated for a strictly TPS-based model, [22] which is rarely used in the healthcare sector, as some adaptation to fit the unique characteristics and circumstances of healthcare is necessary.

The definition of Lean presented in the Background section highlights a continuous improvement culture, empowering people, problem solving, eliminating waste, and standardizing work. While none of these are directly reflected in the names of the factors described above, a closer examination of the 25 items included in the five factors reveals that all these key elements of Lean are represented (Additional file 3). For example, “across my hospital/clinic, leaders at all levels create and sustain an environment of continuous improvement and continuous learning” directly addresses continuous improvement culture, and standard work is captured in a total of four survey items addressing both the front line and senior leadership.

The 5-factor model based on data from HUS showed superior fit by all fit indices compared to the 6-factor model constructed in the earlier stages of LHISI development. Some of these differences may be explained by context –the 6-factor model was based on a dataset from two large academic US hospitals. However, the HUS dataset was also considerably larger (6073 responses vs. 914 responses) making the new factor analyses more robust.

In addition to applicability and validity, the 25 item validated LHISI offers many benefits compared to other existing methods for assessing the progress of Lean implementation in healthcare organizations. The validated LHISI can be launched at any time interval according to the organization’s needs, and it can be directed to all employees or focused on selected departments or units. The original LHISI took short approximately 15 min to complete. Decreasing the number of survey items from 43 to 25 shortens the time required to respond to the survey and may thus help with reaching higher response rates and avoiding survey fatigue among staff members in the future. Furthermore, no site visits or external consultants are required, and surveying all employees gives a 360 degree view into the Lean implementation status rather than relying on interviews with leaders or other representatives only.

The validated LHISI is a practical tool that can help healthcare organizations monitor the progress of their Lean implementation overall and in individual departments or units separately. Organization-wide survey results give an overview on Lean maturity, and changes over time can be used to trace the impact and effectiveness of the organization’s Lean implementation strategy. Benchmarking the LHISI results between individual units may reveal which units are struggling and need support or coaching. This is illustrated in Fig. 2 revealing a difference of 3.2 points in the Daily Management Systemsubscale between the highest performing department (Obstetrics & Gynecology and Pediatrics) and the lowest performing department (surgery) indicating a potential need for an intervention in the latter. Furthermore, the results may help leaders identify model cells and, using the detailed information on different components of Lean implementation by examining the subscale scores and conducting focused gemba visits, best practices that can be spread throughout the organization. Figure 2 indicates that the department of Obstetrics & Gynecology and Pediatrics has reached the highest scores on all subscales and may be a model cell for other clinical departments on the site. Hospital leadership at the HUS Hyvinkää Hospital Area supported two Kaizen events that focused on processes in the Obstetrics & Gynecology and Pediatrics Department with highly successful results that the teams have been able to maintain long-term. The Nurse Director of the department, a trained Lean coach, has played a key role in the department’s successful Lean transformation. The high score in the Daily Management System subscale reflects the disciplined approach to continuous improvement in the department, potentially further enhancing the sustainability of the Kaizen results.

As highlighted by these examples LHISI is a valuable tool for leaders and managers in monitoring the progress of Lean implementation in their organization and making data-based decisions on the implementation strategy.

### Strengths and limitations

One of the major strengths of this study is that data used to establish the validity of the LHISI in the context of Finnish healthcare was collected through a survey sent to all employees of the largest academic hospital system in Finland. Furthermore, careful attention was given to translating the original LHISI into Finnish and Swedish, and experts in Lean management contributed to the translation process to ensure accuracy and integrity of terminology and concepts. Furthermore, the analyses were conducted according to the commonly accepted steps involved in conducting EFA and CFA. The response rate of 23% may be considered a limitation. Due to the large size of HUS, however, the absolute number of responses was adequate for randomly splitting the dataset and conducting both EFA and CFA. The Harman single factor test result was 55% indicating that potential common method bias cannot be ruled out. However, the Harman single factor test has been criticized for its limitations, foremost for being an exploratory method and not a statistical test [25]. The CFA has been suggested a better method as it provides a chi-square test making it possible to judge whether the model fits the data or not. Our CFA revealed that the 5-factor model had a better fit by all measures, including the Chi-square test, than the single-factor model as shown in Table 3. Additionally, since the data used in the analyses were collected in Finland, the results may not be directly generalizable to healthcare organizations operating in other countries and careful attention should be paid to context.

## Conclusions

This study shows that the LHISI is relevant, feasible, applicable, and valid in the context of Finnish healthcare. The main practical implications of our study are that the validated LHISI allows the organization to self-monitor the progress of its Lean implementation and provides detailed actionable information on key Lean elements that can be used to guide decisions on the Lean implementation strategy and development. The most important theoretical implications are that our findings encourage healthcare organizations worldwide to design more studies to use the LHISI in their context and contribute further information to assess its ongoing validity. Cross-national adoption of the LHISI would not only provide a tool for assessing Lean maturity in different healthcare organizations, but also facilitate benchmarking both locally and globally.

## Supplementary Information

**Additional file 1.**

**Additional file 2.** Items removed during Exploratory Factor Analysis.

**Additional file 3.** The 25 survey items of the validated Lean Healthcare Implementation Self-Assessment Instrument (LHISI), version 3.0.

**Additional file 4.** Fit comparison of the 5-factor model with a 6-factor model constructed during earlier stages of LHISI development, and a model with all items loaded on a single factor.

## Data Availability

The datasets used and/or analysed during the current study are available from the corresponding author on reasonable request.

## Abbreviations

CFA: Confirmatory Factor Analysis
CFI: Comparatory Fit Index
CLEAR: Center for Lean Engagement and Research in Healthcare, School of Public Health, University of California, Berkeley
EFA: Exploratory Factor Analysis
HUS: Hospital District of Helsinki and Uusimaa
LHISI: Lean Healthcare Implementation Self-Assessment Instrument
RMSEA: Root mean square error of approximation
SRMR: Standardized root mean square residual

## References

[1] Shortell SM, Blodgett JC, Rundall TG, Kralovec P (2018). Use of lean and related transformational performance improvement Systems in Hospitals in the United States: results from a National Survey. Jt Comm J Qual Patient Saf.

[2] Shingo S (1989). A study of the Toyota production system: from an industrial engineering viewpoint, rev. ed.

[3] Liker J (2004). The Toyota Way. 14 management principles from the world's greatest manufacturer.

[4] Ohno T (1988). Toyota production system: beyond large-scale production.

[5] Womack JP, Jones DT (2003). Lean thinking: banish waste and create wealth in YOur corporation.

[6] Poksinska B (2010). The current state of lean implementation in health care: literature review. Qual Manage Health Care.

[7] Mazzocato P, Savage C, Brommels M, Aronsson H, Thor J (2010). Lean thinking in healthcare: a realist review of the literature. Qual Saf Health Care.

[8] Brandão de Souza L, Pidd M (2011). Exploring the barriers to lean health care implementation. Public Money Manag.

[9] Hasle P, Nielsen AP, Edwards K (2016). Application of lean manufacturing in hospitals-the need to consider maturity, complexity, and the value concept. Hum Factors Ergon Manufacturing Serv Industries.

[10] Mazzocato P, Thor J, Backman U, Brommels M, Carlsson J, Jonsson F, Hagmar M, Savage C (2014). Complexity complicates lean: lessons from seven emergency services. J Health Organ Manag.

[11] Machado Guimarães C, Crespo de Carvalho J (2014). Assessing lean deployment in healthcare—a critical review and framework. J Enterp Transform.

[12] Rundall T, Blodgett J, Reponen E, Shortell S (2020). The lean healthcare implementation self-assessment instrument (LHISI): a principles-based survey instrument to assess lean implementation. Health Serv Res.

[13] R: A language and environment for statistical computing. https://www.R-project.org/. Accessed 2 Nov 2020.

[14] RStudio: Integrated Development Environment for R. https://www.rstudio.com/. Accessed 2 Nov 2020.

[15] psych: Procedures for Personality and Psychological Research. https://CRAN.R-project.org/package=psych Version = 2.0.9. Accessed 2 Nov 2020.

[16] Costello AB, Osborne J (2005). Best Practicers in exploratory factor analysis: four recommendations for getting the Most from Your analysis. Pract Assess.

[17] Worthington RL, Whittaker TA (2006). Scale Development Research:A Content Analysis and Recommendations for Best Practices. Couns Psychol.

[18] Kahn JH (2006). Factor analysis in counseling psychology research, training, and practice:principles, advances, and applications. Couns Psychol.

[19] Rosseel Y (2012). Lavaan: An R package for structural equation modeling. J Stat Softw.

[20] Lt H, Bentler PM (1999). Cutoff criteria for fit indexes in covariance structure analysis: conventional criteria versus new alternatives. Struct Equ Model.

[21] McNeish D, An J, Hancock GR (2018). The thorny relation between measurement quality and fit index cutoffs in latent variable models. J Pers Assess.

[22] Loyd N, Harris G, Gholston S, Berkowitz D (2020). Development of a lean assessment tool and measuring the effect of culture from employee perception. J Manuf Technol.

[23] Vujica Herzog N, Tonchia S (2014). An instrument for measuring the degree of lean implementation in manufacturing. Stroj vestn J Mech E.

[24] Shamah RAM (2013). Measuring and building lean thinking for value creation in supply chains. Int J Lean Six Sigma.

[25] Podsakoff PM, MacKenzie SB, Lee J-Y, Podsakoff NP (2003). Common method biases in behavioral research: a critical review of the literature and recommended remedies. J Appl Psychol.

