# Supplementary material for: Validation of the Lean Healthcare Implementation Self-Assessment Instrument (LHISI) in the finnish healthcare context
Source: BMC Health Serv Res. 2021 Dec 1;21:1289. doi: 10.1186/s12913-021-07322-2 (PMC8638099; doi:10.1186/s12913-021-07322-2)
Supplement: Supplementary file 1 — Additional file 1. [file 12913_2021_7322_MOESM1_ESM.pdf]

Lean Healthcare Implementation Self-Assessment Instrument

(Version 2 – February 2019)

CLEAR
Center for Lean Engagement & Research in Healthcare

©2019 Lean Action Research Learning Collaborative, UC Berkeley

This is an instrument to assess the extent to which lean has been implemented in your healthcare organization, and should take approximately 10 minutes to complete. It was developed by the Center for Lean Engagement and Research (CLEAR) at UC Berkeley, in collaboration with the Lean Action Research Learning Collaborative.

Glossary of Staff Categories

For comparison purposes, the following statements use standardized language. Please review the following terms below, as distinctions may differ from the way you use them within your organization.

Senior Leaders: Hospital or Clinic President, Chief Executive Officer, Chief Operating Officer, Chief Financial Officer, Chief Medical Officer, Chief Nursing Officer, Chief Strategic Officer, and other equivalent titles
Management Staff: Directors, Managers, Supervisors
Clinical Staff: Physicians, Pharmacists, Nurses, Nurse Practitioners, Physician Assistants, Residents, Therapists, Social Workers, Case Managers
Frontline Staff: Medical Assistants, Nursing Assistants, Technicians, and other allied health staff
Administrative and Support Staff: Receptionists, Clerks, Billing Personnel, Environmental Services Personnel, Food Services Personnel, Facilities Personnel, and similar titles
Leaders at All Levels: Senior Leaders and Management Staff

Respondent characteristics

A. Current role (see glossary above):

- Senior Leader
- Management Staff
- Physician
- Nurse
- Frontline Staff
- Administrative and Support Staff
- Performance Improvement Staff

B. Number of years you have worked in this organization: _____

C. In which setting do you primarily work?

- Inpatient
- Outpatient

©2019 Lean Action Research Learning Collaborative, UC Berkeley

For each of the following 43 statements, please rate (on the 0-8 scale) the extent to which it is true within your primary work location and unit or area of responsibility. Choose the selection that most accurately corresponds to your experience and only select "Don't Know" where the meaning is unclear or you do not have any relevant knowledge.

Statement	Never 0	1	2	3	Some-times 4	5	6	7	Always 8	Don't know N/A
1) In my unit/department, goals are visual and understood; everyone knows if goals are being met.	O	O	O	O	O	O	O	O	O	O
2) In my unit/department, management staff use PDSA thinking with the operational units they lead.	O	O	O	O	O	O	O	O	O	O
3) In my unit/department, senior leaders use PDSA thinking with the operational units they lead.	O	O	O	O	O	O	O	O	O	O
4) In my unit/department, employees are provided time and resources for improvement work.	O	O	O	O	O	O	O	O	O	O
5) In my unit/department, senior leaders are committed to lean.	O	O	O	O	O	O	O	O	O	O
6) In my unit/department, management staff are committed to lean.	O	O	O	O	O	O	O	O	O	O
7) In my unit/department, physicians are committed to lean.	O	O	O	O	O	O	O	O	O	O
8) In my unit/department, everyone is empowered and recognized for signaling problems or defects that occur in the area.	O	O	O	O	O	O	O	O	O	O
9) Across my hospital/clinic, leaders at all levels create a safe environment for exposing problems.	O	O	O	O	O	O	O	O	O	O
10) Across my hospital/clinic, senior leaders practice humble inquiry when interacting with employees at all levels of the organization.	O	O	O	O	O	O	O	O	O	O

©2019 Lean Action Research Learning Collaborative, UC Berkeley

Statement	Never<br>0	1	2	3	Some-<br>times<br>4	5	6	7	Always<br>8	Don't<br>know<br>N/A
11) Across my hospital/clinic, leaders at all levels engage employees where the work happens.	O	O	O	O	O	O	O	O	O	O
12) Lean has a sponsor/champion and clinical management staff who demonstrate visible, active, public commitment and support of lean.	O	O	O	O	O	O	O	O	O	O
13) Across my hospital/clinic, leaders at all levels create and sustain an environment of continuous improvement and continuous learning.	O	O	O	O	O	O	O	O	O	O
14) In my unit/department, management staff practice A3 thinking.	O	O	O	O	O	O	O	O	O	O
15) In my unit/department, patient/customer needs drive healthcare work.	O	O	O	O	O	O	O	O	O	O
16) In my unit/department, senior leaders have made an explicit commitment to patient-centered care.	O	O	O	O	O	O	O	O	O	O
17) In my unit/department, frontline staff use visual displays to understand their unit's performance.	O	O	O	O	O	O	O	O	O	O
18) In my unit/department, clinical staff use visual displays to understand their unit's performance.	O	O	O	O	O	O	O	O	O	O
19) In my unit/department, administrative and support staff use visual displays to understand their unit's performance.	O	O	O	O	O	O	O	O	O	O
20) In my unit/department, management staff use visual displays to understand their unit's performance.	O	O	O	O	O	O	O	O	O	O
21) In my unit/department, use of standard work is monitored for compliance.	O	O	O	O	O	O	O	O	O	O
22) In my unit/department, clinical staff use standard work.	O	O	O	O	O	O	O	O	O	O

©2019 Lean Action Research Learning Collaborative, UC Berkeley

Statement	Never 0	1	2	3	Some-times 4	5	6	7	Always 8	Don't know N/A
23) In my unit/department, management staff use standard work.	O	O	O	O	O	O	O	O	O	O
24) In my unit/department, senior leaders use standard work.	O	O	O	O	O	O	O	O	O	O
25) In my unit/department, work processes are standardized.	O	O	O	O	O	O	O	O	O	O
26) In my unit/department, those who provide care to patients/customers communicate with each other.	O	O	O	O	O	O	O	O	O	O
27) In my unit/department, the communication that occurs among those who provide care to patients/customers is focused on problem-solving rather than blaming each other or others.	O	O	O	O	O	O	O	O	O	O
28) In my unit/department, those who provide care to patients/customers share common goals.	O	O	O	O	O	O	O	O	O	O
29) In my unit/department, senior leaders follow a process for strategy definition and deployment that provides focus at all levels.	O	O	O	O	O	O	O	O	O	O
30) Across my hospital/clinic, the outcomes desired from using the lean approach are clear and shared.	O	O	O	O	O	O	O	O	O	O
31) Across my hospital/clinic, the organization's True North vision guides its lean transformation.	O	O	O	O	O	O	O	O	O	O
32) In my unit/department, clinical staff attend daily huddles.	O	O	O	O	O	O	O	O	O	O
33) In my unit/department, management staff attend daily huddles.	O	O	O	O	O	O	O	O	O	O
34) In my unit/department, management staff use value stream mapping.	O	O	O	O	O	O	O	O	O	O

©2019 Lean Action Research Learning Collaborative, UC Berkeley

Statement	Never 0	1	2	3	Some-times 4	5	6	7	Always 8	Don't know N/A
35) In my unit/department, coaching is consistent and evident throughout and at all levels.	O	O	O	O	O	O	O	O	O	O
36) Across my hospital/clinic, leaders at all levels coach to ensure a clear connection between purpose and the work being performed.	O	O	O	O	O	O	O	O	O	O
37) Across my hospital/clinic, leaders at all levels provide employees and staff regular feedback.	O	O	O	O	O	O	O	O	O	O
38) In my unit/department, a daily management system (e.g., daily huddles, gemba walks, etc) is used.	O	O	O	O	O	O	O	O	O	O
39) In my unit/department, management staff review performance data trends to enhance their ability to drive improvement.	O	O	O	O	O	O	O	O	O	O
40) In my unit/department, senior leaders make data driven decisions.	O	O	O	O	O	O	O	O	O	O
41) In my unit/department, clinical and frontline staff use real time, actionable metrics to facilitate problem-solving, problem escalation, and process improvement at all levels.	O	O	O	O	O	O	O	O	O	O
42) In my unit/department, everyone does improvement as part of work, not an extra activity.	O	O	O	O	O	O	O	O	O	O
43) Across my hospital/clinic, successes gained and failures are shared.	O	O	O	O	O	O	O	O	O	O

©2019 Lean Action Research Learning Collaborative, UC Berkeley

Subscale Scoring Rubric

The instrument is designed to incorporate multiple responses from an organization (or unit within an organization), ideally from staff in a range of roles.

Calculate subscale scores by averaging the ratings on the available items listed below within each subscale. We suggest calculating the subscale score only if the respondent has answered at least half of the items included in that subscale. Averaging across the available items will result in a possible range of each subscale from 0-8.

Huddles

32: In my unit/department, clinical staff attend daily huddles.
33: In my unit/department, management staff attend daily huddles.
39: In my unit/department, management staff review performance data trends to enhance their ability to drive improvement.
43: Across my hospital/clinic, successes gained and failures are shared.

Coaching and empowerment

1: In my unit/department, goals are visual and understood; everyone knows if goals are being met.
8: In my unit/department, everyone is empowered and recognized for signaling problems or defects that occur in their area.
12: Lean has a sponsor/champion and clinical management staff who demonstrate visible, active, public commitment and support of lean.
30: Across my hospital/clinic, the outcomes desired from using the lean approach are clear and shared.
35: In my unit/department, coaching is consistent and evident throughout and at all levels.
36: Across my hospital/clinic, leaders at all levels coach to ensure a clear connection between purpose and the work being performed.
37: Across my hospital/clinic, leaders at all levels provide employees and staff regular feedback.
42: In my unit/department, everyone does improvement as part of work, not an extra activity.

Standard work

3: In my unit/department, senior leaders use PDSA thinking with the operational units they lead.
21: In my unit/department, use of standard work is monitored for compliance.
22: In my unit/department, clinical staff use standard work.
23: In my unit/department, management staff use standard work.
24: In my unit/department, senior leaders use standard work.
25: In my unit/department, work processes are standardized.
34: In my unit/department, management staff use value stream mapping.

Commitment

2: In my unit/department, management staff use PDSA thinking with the operational units they lead.
4: In my unit/department, employees are provided time and resources for improvement work.
5: In my unit/department, senior leaders are committed to lean.
6: In my unit/department, management staff are committed to lean.
7: In my unit/department, physicians are committed to lean.

©2019 Lean Action Research Learning Collaborative, UC Berkeley

13: Across my hospital/clinic, leaders at all levels create and sustain an environment of continuous improvement and continuous learning.
14: In my unit/department, management staff practice A3 thinking.

Visual management

17: In my unit/department, frontline staff use visual displays to understand their unit's performance.
18: Clinical staff use visual displays to understand their unit's performance.
19: In my unit/department, administrative and support staff use visual displays to understand their unit's performance.
20: In my unit/department, management staff use visual displays to understand their unit's performance.
38: In my unit/department, a daily management system (e.g., daily huddles, gemba walks, etc) is used.
41: In my unit/department, clinical and frontline staff use real time, actionable metrics to facilitate problem-solving, problem escalation, and process improvement at all levels.

Senior leadership

10: Across my hospital/clinic, senior leaders practice humble inquiry when interacting with employees at all levels of the organization.
16: In my unit/department, senior leaders have made an explicit commitment to patient-centered care.
29: In my unit/department, senior leaders follow a process for strategy definition and deployment that provides focus at all levels.
31: Across my hospital/clinic, the organization's True North vision guides its lean transformation.
40: In my unit/department, senior leaders make data driven decisions.

Communication and trust

9: Across my hospital/clinic, senior leaders at all levels create a safe environment for exposing problems.
11: Across my hospital/clinic, leaders at all levels engage employees where the work happens.
15: In my unit/department, patient/customer needs drive healthcare work.
26: In my unit/department, those who provide care to patients/customers communicate with each other.
27: In my unit/department, the communication that occurs among those who provide care to patients/customers is focused on problem-solving rather than blaming each other or others.
28: In my unit/department, those who provide care to patients/customers share common goals.

©2019 Lean Action Research Learning Collaborative, UC Berkeley
