# Supplementary material for: Validation of the Lean Healthcare Implementation Self-Assessment Instrument (LHISI) in the finnish healthcare context
Source: BMC Health Serv Res. 2021 Dec 1;21:1289. doi: 10.1186/s12913-021-07322-2 (PMC8638099; doi:10.1186/s12913-021-07322-2)
Supplement: Supplementary file 2 — Additional file 2. Items removed during Exploratory Factor Analysis. [file 12913_2021_7322_MOESM2_ESM.docx]

**Additional file 1. Items removed during Exploratory Factor Analysis**

| Item | Step removed |
| --- | --- |
| q03. In my unit/department, senior leaders use PDSA thinking with the operational units they lead. | Item-pair correlation >.8 |
| q05. In my unit/department, senior leaders are committed to lean. | Item-pair correlation >.8 |
| q19. In my unit/department, administrative and support staff use visual displays to understand their units performance. | Item-pair correlation >.8 |
| q20. In my unit/department, management staff use visual displays to understand their units performance. | Item-pair correlation >.8 |
| q23. In my unit/department, management staff use standard work. | Item-pair correlation >.8 |
| q30. Across my hospital/clinic, the outcomes desired from using the lean approach are clear and shared. | Item-pair correlation >.8 |
| q35. In my unit/department, coaching is consistent and evident throughout and at all levels. | Item-pair correlation >.8 |
| q01. In my unit/department, goals are visual and understood; everyone knows if goals are being met. | Cross-loaded item with the lowest high loading (Round 1) |
| q42. In my unit/department, everyone does improvement as part of work, not an extra activity. | Cross-loaded item with the lowest high loading (Round 2) |
| q34. In my unit/department, management staff use value stream mapping. | Cross-loaded item with the lowest high loading (Round 3) |
| q17. In my unit/department, frontline staff use visual displays to understand their units performance. | Cross-loaded item with the lowest high loading (Round 4) |
| q31. Across my hospital/clinic, the organizations True North vision guides its lean transformation. | Cross-loaded item with the lowest high loading (Round 5) |
| q18. In my unit/department, clinical staff use visual displays to understand their units performance. | Cross-loaded item with the lowest high loading (Round 6) |
| q39. In my unit/department, management staff review performance data trends to enhance their ability to drive improvement. | Cross-loaded item with the lowest high loading (Round 7) |
| q08. In my unit/department, everyone is empowered and recognized for signaling problems or defects that occur in their area. | Cross-loaded item with the lowest high loading (Round 8) |
| q15. In my unit/department, patient/customer needs drive healthcare work. | Cross-loaded item with the lowest high loading (Round 9) |
| q41. In my unit/department, clinical and frontline staff use real time, actionable metrics to facilitate problem-solving, problem escalation, and process improvement at all levels. | Cross-loaded item with the lowest high loading (Round 10) |
| q04. In my unit/department, employees are provided time and resources for improvement work. | Cross-loaded item with the lowest high loading (Round 11) |
