# Supplementary material for: Validation of the Lean Healthcare Implementation Self-Assessment Instrument (LHISI) in the finnish healthcare context
Source: BMC Health Serv Res. 2021 Dec 1;21:1289. doi: 10.1186/s12913-021-07322-2 (PMC8638099; doi:10.1186/s12913-021-07322-2)
Supplement: Supplementary file 3 — Additional file 3. The 25 survey items of the validated Lean Healthcare Implementation Self-Assessment Instrument (LHISI), version 3.0. [file 12913_2021_7322_MOESM3_ESM.docx]

**Additional file 3. The 25 survey items of the validated Lean Healthcare Implementation Self-Assessment Instrument (LHISI), version 3.0**

| Survey Items |
| --- |
| *Leadership* |
| 1. Across my hospital/clinic, leaders at all levels create a safe environment for exposing problems. |
| 2. Across my hospital/clinic, senior leaders practice humble inquiry when interacting with employees at all levels of the organization. |
| 3. Across my hospital/clinic, leaders at all levels engage employees where the work happens. |
| 4. Across my hospital/clinic, leaders at all levels create and sustain an environment of continuous improvement and continuous learning. |
| 5. In my unit/department, senior leaders have made an explicit commitment to patient-centered care. |
| 6. In my unit/department, senior leaders follow a process for strategy definition and deployment that provides focus at all levels. |
| 7. Across my hospital/clinic, leaders at all levels coach to ensure a clear connection between purpose and the work being performed. |
| 8. Across my hospital/clinic, leaders at all levels provide employees and staff regular feedback. |
| 9. In my unit/department, senior leaders make data driven decisions. |
| 10. Across my hospital/clinic, successes gained and failures are shared. |
| *Commitment* |
| 11. In my unit/department, management staff use PDSA thinking with the operational units they lead. |
| 12. In my unit/department, management staff are committed to lean. |
| 13. In my unit/department, physicians are committed to lean. |
| 14. Lean has a sponsor/champion and clinical and management staff who demonstrate visible, active, public commitment and support of lean. |
| 15. In my unit/department, management staff practice A3 thinking. |
| *Standard work* |
| 16. In my unit/department, use of standard work is monitored for compliance. |
| 17. In my unit/department, clinical staff use standard work. |
| 18. In my unit/department, senior leaders use standard work. |
| 19. In my unit/department, work processes are standardized. |
| *Communication* |
| 20. In my unit/department, those who provide care to patients/customers communicate with each other. |
| 21. In my unit/department, the communication that occurs among those who provide care to patients/customers is focused on problem-solving rather than blaming each other or others. |
| 22. In my unit/department, those who provide care to patients/customers share common goals. |
| *Daily Management System* |
| 23. In my unit/department, clinical staff attend daily huddles. |
| 24. In my unit/department, management staff attend daily huddles. |
| 25. In my unit/department, a daily management system (e.g., daily huddles, gemba walks, etc) is used. |
