# Supplementary material for: Validation of the Lean Healthcare Implementation Self-Assessment Instrument (LHISI) in the finnish healthcare context
Source: BMC Health Serv Res. 2021 Dec 1;21:1289. doi: 10.1186/s12913-021-07322-2 (PMC8638099; doi:10.1186/s12913-021-07322-2)
Supplement: Supplementary file 4 — Additional file 4. Fit comparison of the 5-factor model with a 6-factor model constructed during earlier stages of LHISI development, and a model with all items loaded on a single factor. [file 12913_2021_7322_MOESM4_ESM.docx]

**Additional file 4. Fit comparison of the 5-factor model with a 6-factor model constructed during earlier stages of LHISI development, and a model with all items loaded on a single factor.**

| Fit index | 5-factor model | 6-factor model | Single factor model |
| --- | --- | --- | --- |
| Chi square (df), p-value | 4013.756 (265), 0 | 10018.448 (512), 0 | 28154.845 (860), 0 |
| CFI | 0.921 | 0.868 | 0.705 |
| TLI | 0.911 | 0.855 | 0.69 |
| AIC | 215342.435 | 287074.112 | 375421.148 |
| RMSEA | 0.068 | 0.078 | 0.102 |
| SRMR | 0.05 | 0.068 | 0.073 |

Abbreviations: AIC, Aikake’s information criterion; CFI, comparative fit index; df, degrees of freedom; RMSEA, root mean square error of approximation; SRMR, standardized root mean square residual; TLI, Tucker-Lewis Index
